# Steroids and L-Lysine Aescinate for Acute Radiculopathy Due to a Herniated Lumbar Disk

**DOI:** 10.3390/medicina55110736

**Published:** 2019-11-14

**Authors:** Mykhaylo Oros, Mykhailo Oros Jar, Vasyl Grabar

**Affiliations:** SHEI, Uzhhorod National University, Uzhhorod 88000, Ukraine; mihoros@meta.ua (M.O.); orosmisha13@gmail.com (M.O.J.)

**Keywords:** discogenic radiculopathy, sciatica, therapy, glucocorticoids, L-lysine aescinate

## Abstract

Background and objectives. The efficacy of commonly prescribed analgesic and adjuvant drugs for the management of patients with radiculopathy has not been well established. Oral steroids are commonly used to treat sciatica or radiculopathy due to a herniated disk but the effect remains controversial. L-lysine aescinate showed superiority over placebo or baseline therapy with NSAIDs alone in treating sciatica, but have not been evaluated in an appropriately powered clinical trial. Materials and Methods. Randomized, double-blind clinical trial conducted in two health centers in collaboration with Uzhhorod Natioanl University in Ukraine. Adults (N = 90) with acute radicular pain and a herniated disk confirmed by MRI were eligible. Participants were randomly assigned to three groups (N = 30 in each) to receive a baseline therapy with lornoxicam (16 mg per day) and adjunctive 5-day course of IV dexamethasone (first group: 8 mg per day/40 mg total) or 0,1% solution of L-lysine aescinate (5 mL and 10 mL for group 2 and 3 respectively). Primary outcomes were Visual Analogue Scale changes and the straight leg raise angle at 15th and 30th day. Results. The level of pain improvement at 15th days after initiation of therapy with dexamethasone or solution of L-lysine aescinate at doses of 5 or 10 mL was not significantly different. The lowest levels of pain were achieved in patients who received the L-lysine aescinate 10 mL, but the range of decrease in pain was slightly greater in the group administered dexamethasone. Conclusions. Among patients with acute radiculopathy due to a herniated lumbar disk a short course of IV dexamethasone or L-lysine aescinate resulted in pain improvement at 15th and 30th day. Dexamethasone may be preferable if a longer-term analgesic effect is needed. Taking into account side effects of dexamethasone, a solution of L-lysine aescinate can be used to relieve pain symptoms.

## 1. Introduction

Low back pain is an extremely common and important health problem worldwide [1,2,3,4]. In 2015, over half a billion people worldwide had low back pain of more than three months duration [5,6]. It is the leading cause of activity limitation and one of the most common conditions for which patients seek medical care [7,8,9,10].

Low back-related leg pain is one of the most common variations of back pain, with about two thirds of patients presenting with it [11,12,13]. Extensive work has been published on classification systems, where researchers have attempted to optimize management and improve outcomes by subgrouping patients with low back pain into homogeneous populations with similar characteristics. Leg pain can be classified as either radicular pain due to spinal nerve root involvement, or non-specific pain due to back pain that spreads down the leg from structures such as ligaments or joints, but not involving a spinal nerve root [14]. A range of definitions and terms are used to describe pain due to spinal nerve root involvement including sciatica, radicular pain or radiculopathy, and various diagnostic criteria are used to define it [15,16,17,18,19,20]. The majority of published guidelines on low back pain propose identifying patients with leg pain thought to be due to nerve root involvement, because treatment options and management for this condition may be different from those for non-specific pain. Sciatica is considered to be a prognostic indicator of poor outcome among patients with low back pain. Patients with a clinical diagnosis of sciatica are about five times more likely to take drugs than those with low back pain only [21,22,23,24].

While guidelines provide clear and consistent recommendations for the prescription of drugs for non-specific low back pain, this is not the case for sciatica or radicular pain. Drugs commonly prescribed for the management of sciatica include non-steroidal anti-inflammatory drugs, skeletal muscle relaxants, systemic corticosteroids, antidepressants, and anticonvulsants [25,26,27]. At present, the efficacy and tolerability of commonly prescribed analgesic and adjuvant drugs for the management of patients with sciatica has not been well established. Evidence from a few trials and meta-analysis provides limited support for the use of NSAIDs and corticosteroids to relieve pain in the short term in patients with acute sciatica.

Complementary and adjunctive medicine is widely applied in clinical practice. Practice routine of primary care physicians and some specialized care centers shows some support for the use of L-lysine aescinate. However, the use of this agent in patients with radiculopathies and back pain is currently not recommended in clinical guidelines. However, there are few clinical trials and a low-level of evidence due to the poor study design or small sample size. As most guidelines recommend a course of conservative care before surgery is considered, it is imperative to understand what best practice conservative care should entail [25,28,29,30].

The available evidence does not clearly show favorable effects of analgesics, corticosteroids, antidepressants, or anticonvulsants, even compared with placebo. New strategies could be developed in expecting to improve pain management in the proportion of people with sciatica and radiculopathy. The purpose of this study was to test the effects of a IV dexamethasone or L-lysine aescinate on symptoms of acute radiculopathy and sciatica in a clinical trial.

## 2. Methods

To address this issue, we performed a parallel-group, double-blind randomized clinical trial of a course of dexamethasone vs. L-lysine aescinate for patients with an acute lumbar radiculopathy associated with a herniated lumbar disk. The study was conducted among the “Vodoliy” Clinic (Khust, Ukraine), which collaborates with the Uzhhorod National University, and the Regional Clinical Center of Neurosurgery and Neurology (Uzhhorod, Ukraine) from April to July 2018. The local ethics committee approved the study protocol (Bioethics Commissions of Uzhhorod National University, ethical review of research protocol# 6, approval date 05 April 2018.). 

The study included patients aged 18 to 75 years with a primary or recurrent episode of sciatica, lasting from one to four weeks. Sciatica or radiculopathy was defined as pain in the leg (with or without lumbar pain) spreading below the knee in the radicular pattern, with signs of root irritation (a positive straight leg test), and/or radicular neurological deficit (sensory or reflex). Additionally, the inclusion criterion was neuroimaging—MRI with the presence of protrusion of the intervertebral disc that correlate with the level of clinical manifestations.

Exclusion criteria: contraindications for use of corticosteroids or L-lysine aescinate, onset of radicular pain more than two weeks prior, previous lumbar surgery, substantial or progressive motor loss, diabetes mellitus, and severe somatic pathology (uncontrolled arterial hypertension, renal, hepatic, or heart failure), systemic or epidural use of corticosteroids during the last six weeks, and pregnancy.

Description of Participants and Study Procedures. We assessed 94 patients for eligibility; four were excluded or declined to participate (one patient withdrew consent before receiving the IV bolus, and three refused subsequent evaluations). Patients who met the criteria (total *N* = 90) were randomized to three groups, comparable by the number of participants and baseline characteristics (Table 1). After providing informed consent, patients were reviewed for eligibility by a spine-specialist physician. In all patients, MRI was performed before the start of treatment and pain intensity was determined using Visual Analog Scale (VAS) and Brief Pain Inventory (BPI, and the straight leg test and *N*-reflex measure were performed according to standard methods.

During the first day of the study, no drugs were used. From the second day, all patients received equivalent conservative therapy that included analgesic and nonsteroidal anti-inflammatory therapy of 16 mg of lornoxicam per day.

The first group received a solution of dexamethasone, the second group received a 0.1% solution of L-lysine aescinate 5 mL, and the third received a 0.1% solution of L-lysine aescinate 10 mL. Drugs were injected intravenously once a day, the course of treatment was five days. Information about the drug was hidden from patients, thus the study was blind.

In the first group, patients received an additional therapy, IV dexamethasone solution (intravenous bolus at a dose of 8 mg per day), for five days. Patients from the other two groups, besides the lornoxicam, received a solution of L-lysine aescinate 1 mg in 1 mL (0.1% solution) in doses of 5 mL and 10 mL per day, respectively, in the form of intravenous infusion with dilution with an isotonic solution of sodium chloride to 50 mL of total (also for five days).

On the first day, a magnetic resonance examination of the lumbar-sacral spine was carried out as well as a Visual Analogue Scale for pain (VAS; 0–10), the measurement of the pain intensity and functional significance were tested with the Brief Pain Inventory (Short Form). The intensity of the straight leg test (a lifting test of the straightened leg) was determined with the fixation of results from 30° to 70°. In patients with clinical or visualization signs of the S1 root injury electroneuromyography was additionally performed to measure the amplitude of the H-reflex. The Brief Pain Inventory and amplitude of the H-reflex were tested as potential prognostic markers and predictors of sciatica or radiculopathy course.

The results were evaluated on the fifteenth and thirtieth days of the study. On the fifteenth and thirtieth day after the start of treatment, symptom improvement was assessed using VAS and a measurement of the intensity of the straight leg test as well as repeated MRI.

The control points were defined in order to determine the probability of improving the functional state of patients in the early period when the symptoms were most intense as well as in the time interval defined by the guidelines as an indication of surgical intervention in the case of ineffectiveness of conservative measures (duration of pain for 6–8 weeks).

Statistical Analysis. The dynamic of VAS levels, the straight leg test, differences between the groups as well as the possible prediction of the course by BPI and the amplitude of the H-reflex were statistically analyzed in the software environment R version 3.4.3. Since the research design was hierarchical (in each of the three groups, grouped repeated measurements from one and the same patient), and there were also factors with fixed effect (fixed effect, the patient took the medication, an assessment on the short questionnaire of pain, the intensity of the H-reflex) and random effect (random difference); for the analysis, the method of linear mixed regression models (mixed models) was used. For the construction of mixed regression models, an additional software package nlme 3.1-131 was used. The normal distribution of the residue of the regression model and the absence of statistical emissions were verified by inspection of a quantile–quantile normal graph. The statistical significance of the influence of factors (BPI and H-reflex) on the dynamics of pain in patients was determined by the dispersion analysis of regression models (one contained a possible influence factor, the other one was not). A group of patients treated with dexamethasone was used as the reference group for comparison. It is worth noting that repeat measurements in patients are not expected to be independent of each other. The patient’s condition on the fifteenth and thirtieth day depends to a certain extent on its initial state. Therefore, in order to take into account the time communication between successive measurements of the same patient, we examined the expediency of correction of temporal autocorrelation in a mixed regression model. For this, the likelihood models of the first-order auto-recursive component were compared using the Akaike information criterion (AIC) and likelihood ratio test. Statistically significant results were considered with *p* < 0.05. The dynamics of the patient’s condition was visualized using Sankey diagrams.

## 3. Results

### 3.1. Dynamic of the Pain by Measuring the Visual Analog Scale

**Figure 1:**
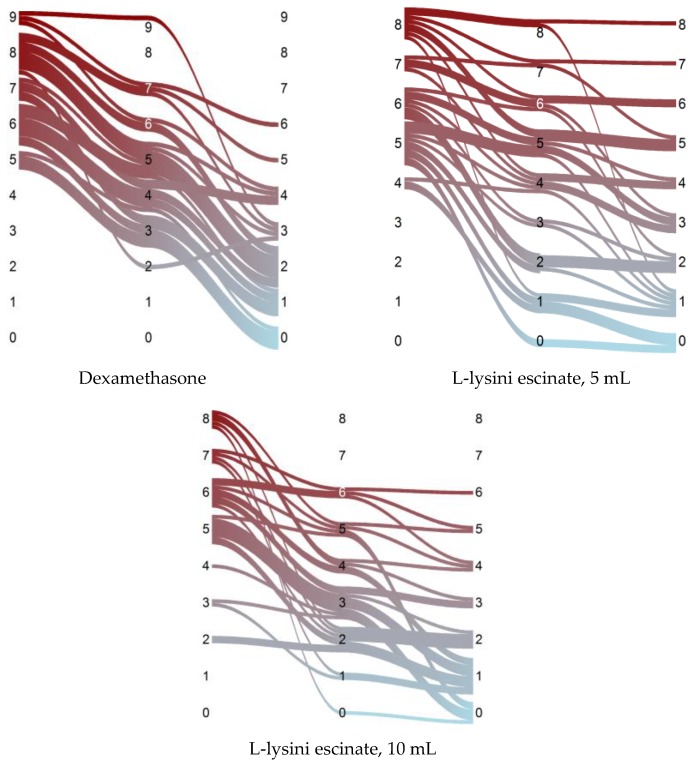
The dynamics of the patient outcome by the Visual Analog Scale. Vertical coordinates correspond to the VAS, the flow width is proportional to the number of patients.

Baseline intensity of pain on the VAS scale was 6.9 ± 1.3 points in the first group, and exceeded similar rates in the second and third groups (in the second group, the average value of VAS was 6.1 ± 1.3 points, in the third −5.7 ± 1.7 points (Table 1). On the fifteenth day, from the onset of treatment, improvement in condition and reduction in pain were recorded in all groups. Only few patients did not react to therapy, maintaining the same level of pain as before treatment (Figure 1).

**Figure 2:**
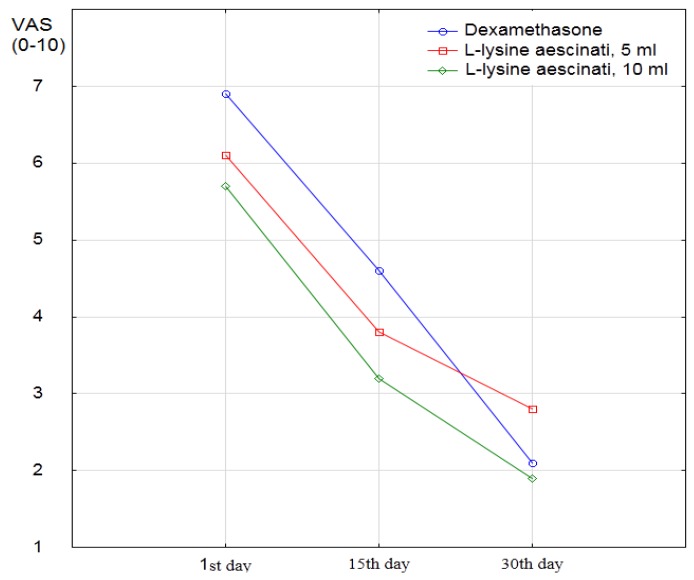
The dynamic of the Visual Analog Scale evaluated on the 1st, 15th, and 30th days of the study.

On the thirtieth day after initiated treatment, the average intensity of pain continued to decrease in all groups; the lowest level of pain on the thirtieth day was achieved in patients who received the drug L-lysine escinate 10 mL, but the range of the decrease in pain was slightly better in the dexamethasone group (Figure 2).

The mixed regression model also showed the initial difference between the groups according to the intensity of pain, and this difference in the case of the third group reached the statistical significance (*p* = 0.051 and *p* = 0.0050 for comparison of the second and third groups with the first one). The effect of this initial difference on the results of the comparison was corrected by inclusion of the absolute data obtained in each of the successive measurements into the model. The dynamics of pain reduction on the VAS scale on the fifteenth day lost statistical significance and reached *p* = 0.15, and only the effect on the thirtieth day retained statistical significance with *p* = 0.012.

Reducing the intensity of pain on the VAS scale after the course of dexamethasone is statistically significant at the fifteenth (average decrease of 2.30 points, *p* < 0.0001) and thirtieth day (mean decrease of 4.83 points, *p* < 0.0001). On the fifteenth day, the decrease in the score of the VAS scale in both groups of L-lysine aescinate (5 mL and 10 mL) did not differ from the effect of dexamethasone (*p* = 0.93 and *p* = 0.75, respectively). However, already on the thirtieth day in the second group, the reduction was weaker on average by 1.47 points on the scale of VAS (*p* = 0.0038). In the third group (compared with the first group), the decrease on the scale of VAS was weaker on average by 1.07 points (*p* = 0.040).

The short-term effect was the same for both L-lysine groups in comparison with the dexamethasone group. Moreover, in the case of the L-lysine aescinate, effect change with the dosage increasing was very weak. Therefore, it will be more rational to use exactly 5 mL of this drug. Concerning the same long-term goal of reducing the intensity of pain, the course of dexamethasone showed a greater effect than the L-lysine aescinate.

### 3.2. The Brief Pain Inventory and the Amplitude of the H-Reflex as Potential Prognostic Markers and Predictors of Sciatica or Radiculopathy Course

To the mixed regression model, as a possible modifying factor or predictor of patient dynamics, an assessment of the pain intensity for the Brief Pain Inventory before treatment was included. One of the patients was not evaluated by the respondent; the data was excluded from the analysis. The new model was characterized by the parameter AIC = 1022, compared to an AIC = 1033 in the model without the BPI factor. Additionally, the likelihood ratio of these models was LR = 20.8, and *p* = 0.00090. Thus, the initial assessment of BPI had an effect or correlated with the intensity of pain on the VAS scale with a statistical significance. The analysis of the coefficients of the mixed regression model (Table 2) indicates that we can deal with both the correlation for BPI and VAS, and with the effect on the course of treatment.

The value of the coefficient for BPI in the first day βKOБ-1 = 0.077 means that at the beginning of treatment, every 10 points on the BPI scale were associated with an increase in VAS by 0.77 points. It is clear that both scales measured the pain-related problems, and the correlation between them was expected.

The model explains the stronger positive dynamic of participants who had high initial values on the BPI scale. That is, if the stronger dynamics in some patients is explained not by the effect of drugs, but their initially worse condition in accordance with the scale of BPI, then the calculated factor of the influence of drugs is reduced. This means that patients with high initial BPI demonstrate a further, and even more rapid decrease in pain intensity in the interval between the fifteenth and thirtieth day. Patients with high initial levels on the BPI scale will have a better additional positive dynamic.

As a possible predictive factor of the course of back pain, the H-reflex was also measured. The factor of the H-reflex was introduced into a mixed regression model. The information criterion for the AIC model with the H-reflex was AIC = 1035, whereas in the similar model without the introduction of the H-reflex, the AIS = 1029. The statistical significance when comparing these two models was *p* = 0.59, and it should be understood that this statistical significance relates to the hypothesis that the simpler model without a H-reflex dominates the complicated model. Therefore, consideration of the assessment of the H-reflex in the study and description of the dynamics of the pain symptom in the studied patients is not appropriate.

### 3.3. Dynamics of the Straight Leg Test

The straight leg test was performed and measured before starting the treatment, on the fifteenth and thirtieth day after starting treatment. Before analyzing the results from the data array, patients who had a negative straight leg test symptom prior to treatment and patients with a pseudosymptomatic straight leg test (one patient in the first group) were excluded.

**Figure 3:**
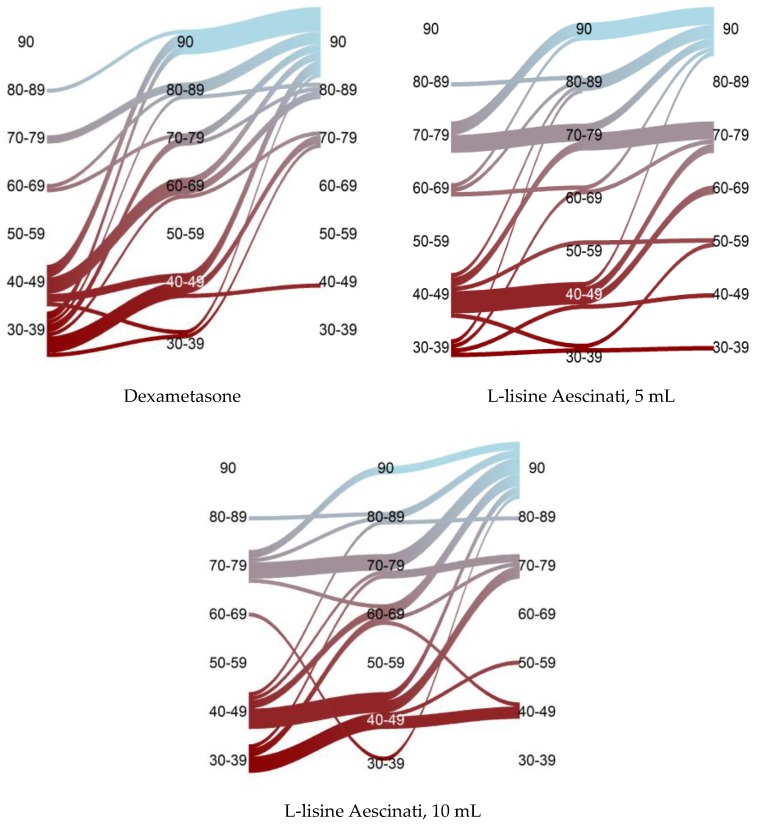
Dynamics of the patients of the three groups in a degree of the symptom of straight leg test. Vertical coordinates correspond to the assessment of the straight leg test, a number (90 indicates a negative symptom of straight leg test) of patients with an initial negative symptom of straight leg test are excluded from the analysis, the width of the streams is proportional to the number of patients.

The significance of the straight leg test symptom for those patients who achieved a negative symptom as a result of the received pharmacotherapy was taken into account in the analysis as 90°. At the beginning of the experimental study, the mean values of the straight leg test symptom in the first group were 42.8° ± 14.2°, which was slightly less than in the second group (52.8° ± 15.6°) and the third group (50.6° ± 17.7°). At the same time, on the fifteenth day, the mean values of the Laseg’s symptom in the first group (65.2° ± 20.2°) were slightly lower than those in the second (64.4° ± 19.4°) and the third (60.8° ± 17.1°) groups. This difference increased even more on the thirtieth day. Additionally, on the thirtieth day after the start of the trial, the proportion of patients with a negative symptom reached 67% in the first group, 67% in the second group, and 60% in the third group (Figure 3).

**Figure 4:**
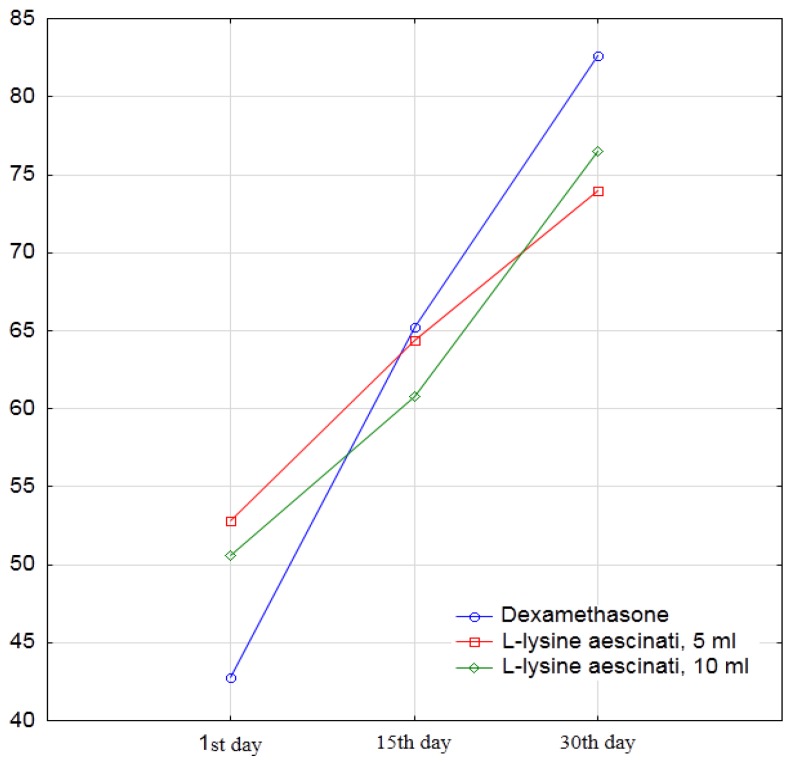
The dynamic of the straight leg raise angle on the 1st, 15th, and 30th days of the study.

The regression coefficient of the mixed model confirms the growth of the degree of the straight leg test in the first group on average by 22.4° on the fifteenth day and by 39.8° on the thirtieth day. Patients in the second and third groups had better initial values for the symptoms of the straight leg test (averagely 10.0° and 7.8°, respectively), but weaker than the positive dynamics. On the thirtieth day, the overall positive dynamics of patients receiving L-lysine aescinate was weaker by an average of 18.3° in the case of a dose of 5 mL (*p* = 0.0015) and 13.9° (*p* = 0.014) in the case of dosing 10 mL, than in patients receiving dexamethasone (Figure 4).

**Figure 5:**
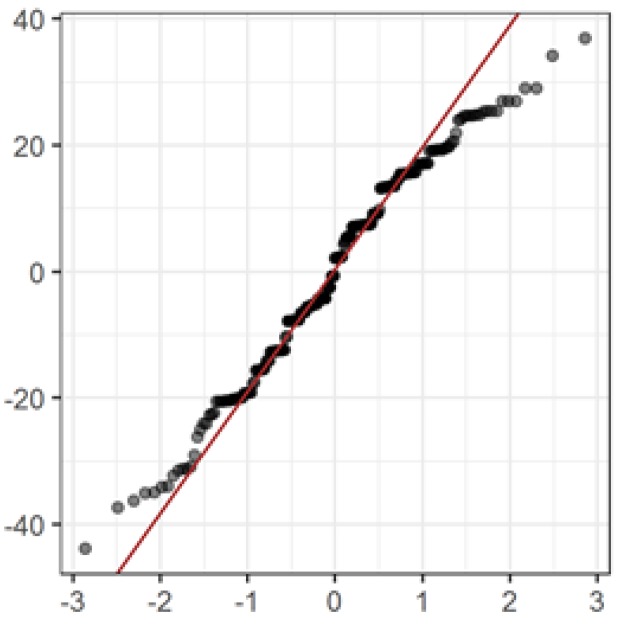
Quantum–quantile normal graph of residues of a mixed regression model, evaluated by degree of the straight leg test.

The verification of the normal distribution of residues of a mixed regression model indicates some deviations (Figure 5).

This may be due to a fairly low accuracy and stability when measuring the straight leg test symptom, and also the existence of an upper threshold (a negative straight leg test is often present in our data). In general, this reduces the reliability and the value of the mixed regression model presented here.

## 4. Discussion and Clinical Cases

To our knowledge, this is the first trial of L-lysine aescinate in acute discogenic sciatica with a significant sample size. Additionally, it seems to be the first trial of dexamethasone not used as an emergency one-time bolus, but as a course for five days. We found a significant improvement of sciatic pain with dexamethasone and L-lysine aescinate. According to our results, the lowest levels of pain on the thirtieth day were achieved in patients who received the drug L-lysine aescinate 10 mL, but the range of decrease was slightly greater in the group of dexamethasone. This means that dexamethasone is effective for pain relief in patients with sciatica and may be preferable if a longer-term effect is needed. Taking into account the side effects of dexamethasone, a solution of L-lysine aescinate can be used to relieve pain symptoms. The effect change is insignificant with the dosage increase of L-lisine aescinate and the short-term effect is the same for both L-lysine groups in comparison with the dexamethasone group.

Brief Pain Inventory values correlated with the assessment of pain by the VAS and straight leg test, which is logically explained by a common description of pain characteristics. At the beginning of treatment, every 10 points on the BPI scale was associated with a VAS increase of 0.77 points. Patients with high baseline BPI points will have better additional positive dynamics on the fifteenth day. Namely, they will observe VAS indexes on the fifteenth day below the average of 0.21 points for every 10 points of the initial BPI value (after taking into account the effect of the drug). Patients with high initial BPI demonstrate a further, and even more rapid decrease in the intensity of pain in the interval between the fifteenth and thirtieth day. If the BPI factor and a certain role in the model of the dynamics of the straight leg test are assigned, then this role is limited to the correlation of the intensity of pain on the BPI scale and degrees (the symptom is more pronounced in patients with higher BPI scores).

The degree of the H-reflex, measured before the start of treatment, did not affect the subsequent dynamics of the patients. The significance of the H-reflex in the study is not appropriate since the statistical significance when comparing the two models with the dispersion analysis was *p* = 0.59. Similar results were obtained in the study of the H-reflex as a potential predictor in a mixed model with a degree of the straight leg test symptom. Therefore, the possible influence of the degree of the H-reflex on the future dynamics of the degree of the straight leg symptom under the conditions of the performed exertion has not been confirmed (LR = 5.2, *p* = 0.59).

Our results are consistent with previous studies. Over the past 40 years, no more than ten comparable studies have been published on the efficacy of systemic corticosteroids for sciatica and radicular pain [31,32,33,34,35,36,37,38]. Some studies have shown a tendency for improvement in pain and a significant improvement in functioning after one month after single administration of methylprednisolone [36]. The single intravenous administration of dexamethasone also showed some effect in improving the pain and increasing the volume of movement in patients with radiculopathy, although the authors noted that the effect was short-lived and the pathogenetic mechanism of analgesia was not completely understood [38]. Course use of prednisolone in the acute period showed a statistically significant improvement of the function, but without significant pain relief [37].

The above studies have certain disadvantages, in particular, some of them were not statistically significant, for example, due to the small number of patients [32,33,35]. One-time administration of drugs is noteworthy, but it is unlikely to produce a lasting effect [38], while pulse therapy raises many questions about safety and justification with regard to the severity of the condition [36]. In most of the course trials, prednisolone or methylprednisolone, known for its dose-dependent and somewhat postponed effects, is often indicated by the authors themselves [37].

In search of new ways and effective means of treating patients with radiculopathies, it was decided to explore the drug L-lysine aescinate. The background is based on the polymodal effect of the drug. L-lysine aescinate reduces the activity of lysosomal hydrolases, thereby inhibiting the decrease of glycosaminoglycans, which in clinical practice manifests as membrane-stabilizing and anti-exudative effects, and the inhibition of phospholipase A2 with moderate anti-inflammatory action [39,40,41,42,43].

Studies of the efficacy of this agent in radiculopathies and sciatica showed superiority over placebo or “baseline therapy” [44,45,46,47]. However, it should be noted that the studies were evaluated only for acute periods and had a moderate clinical significance, although they reached statistical significance. In our study, for the first time, the effectiveness of L-lysine aescinate in different doses versus dexamethasone was compared. This task was justified by the similarity of influence on the main links of pathogenesis: the anti-inflammatory and anti-edema effect of corticosteroids is considered as a potential mechanism of positive action in radiculopathies.

Our study used a short course of dexamethasone, which was more justified in terms of the pathophysiology of the process and clinical needs (rapid effect and favorable side effects profile). The level of pain improvement at 15 days after the initiation of therapy with 0.4% dexamethasone in 2 mL or 0.1% solution of L-lysine aescinate at doses of 5 or 10 mL was not significantly different. The difference in dynamics for 30 days was 1.47 ± 0.50 points on the VAS scale when compared to dexamethasone with 0.1% solution of L-lysine aescinate in a dose of 5 mL (*p* = 0.0038) and 1.07 ± 0.52 scores when comparing dexamethasone with a 0.1% solution of L-lysine aescinate in a dose of 10 mL (*p* = 0.040).

In a prospective study that included 206 patients, Dillingham and colleagues evaluated electrophysiological examination and surgical confirmation of the intervertebral disk hernia [48]. The authors found that an EMG study allowed for the determination of 97% of radiculopathies and 89% of surgically confirmed hernias. Marin and colleagues conducted research to identify more specific signs of radiculopathy and diagnostic value for some of them [49] and the results showed that the sensitivity of the H-reflex to detect radiculopathy L5 was only 6%, S1 to 50% with a specificity of 91%. The H-reflex is considered by the North American Society of Spinal Surgery as an additional survey, which confirms the radiculopathy S1 with the hernia of the intervertebral disc [27], and has the greatest predictive value [50].

According to our results, the size of the intervertebral disk herniation, the degree of the straight leg test, BPI, and the H-reflex does not have a reliable prognostic value and does not allow for predicting the course of the radiculopathy, in particular the regression of pain or need for the future surgical treatment. Therefore, only a thorough repeated evaluation of clinical manifestations and the presence of “red flags” is a reliable indicator of the negative course of sciatica with radiculopathy and may serve as the basis for changing the method of treatment, in particular, surgical.

The morphometric characteristics of the intervertebral disc according to MRI do not correlate with the intensity of pain and are not valid predictors of the clinical condition. The predictive value of the Brief Pain Inventory and the straight leg test requires further research.

### 4.1. Clinical Cases and Progression of Radiculopathies Associated with Intervertebral Disk Herniation

Although the exact mechanism of the natural course of radiculopathy associated with the vertebral hernia are not fully understood, it is considered that the condition of most patients will improve regardless of the method of treatment [27].

Teplick and Haskin, for more than 30 years ago, first described the spontaneous regression of the herniated disk in 11 patients. Since then, many cases have been documented with confirmation of repetitive CT and MRI scans [51,52,53,54]. The literature proposes three possible mechanisms for this phenomenon. The first is the re-retraction of extruded material into the intervertebral space. The second one is based on the phenomena of dehydration and “shrinkage” of the hernia. The most modern theory believes that enzymatic degradation, inflammation due to cytokines, and metalloproteinase with subsequent phagocytosis, leads to reabsorption [55,56,57,58].

### 4.2. Clinical Case 1 (Dexamethasone Group)

Patient M., 64 years old, complains of intense back pain with spreading to the left leg, tingling in the foot. Clinically: VAS 8. The straight leg test is left-angled to 30°. Achilles reflex is absent left-sided, hypoesthesia in the dermatome S1.

**Figure 6:**
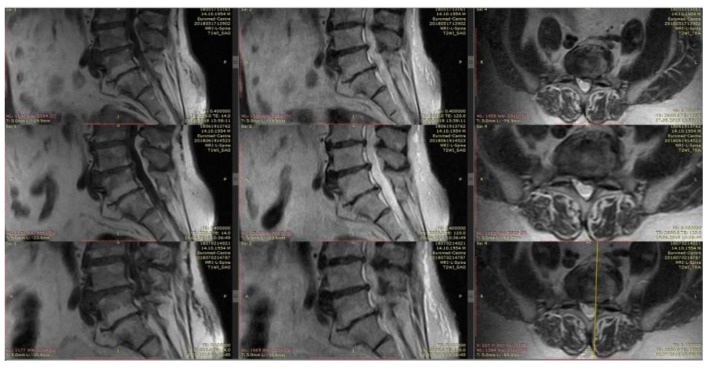
MRI of the lumbar spine images show a large extruded disc fragment at the L5-S1 level. Neuroimaging for 15 and 30 days does not indicate changes of the size of extrusion.

Initial MRI: The extrusion of the intervertebral disk L5–S1 up to 8 mm with stenosis of the foraminal channel is left-sided and compressed by the corresponding structures. With the treatment of dexamethasone, already on day 15 (the first control point), the VAS was 1–2 points, the volume of movements in full. However, neuroimaging for 15 and 30 days does not indicate changes in the intensity of closure plates (type Modic II) or the size of the hernia (Figure 6).

### 4.3. Clinical Case 2 (L-Aesineate 5 mL Group)

In our opinion, this case needs an additional comment. It is generally considered that an increase in the size of the intervertebral disk hernia is a negative predictor, but the data from numerous publications and observations indicate the opposite. Intervertebral hernia accompanied by sequestration or extrusion, without motor deficiency clinically, is more likely to by reabsorbed and “shrink”. Despite the large size, it is easier to dehydrate and reconfigure them in such a way that they do not create compressions of neuronal structures [59,60].

Patient M., 41, complains of moderate back pain and “striking” pain in the right leg. At the primary examination: excess body weight, neurological status without sensory, reflex, or motor deficiency. VAS: 5 points. Straight leg test right-side to 35°.

MRI: On the background of degenerative-degenerative changes and protrusions, there is a L4–L5 level hernia. At the first control, extrusion of bulging and caudal migration.

**Figure 7:**
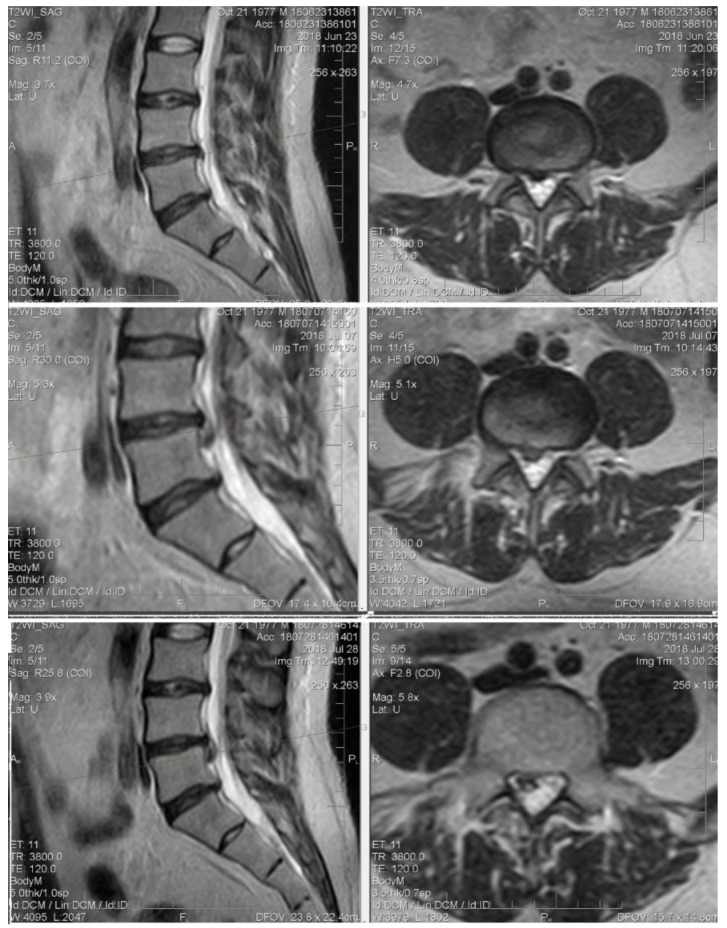
Initial T2-weighted MRI of the lumbar spine shows a large extruded disc at the L4-L5 level and the follow-up imaging shows up migrating fragment.

It is interesting that the VAS values at the first control was 0 points. In this case, the increase in size and shape changes were an element of sanogenesis: caudally displaced hernia does not compress neuronal structures (Figure 7).

### 4.4. Clinical Case 3 (L-Lysine Group, 10 mL)

An indicative case that confirms the thesis “big hernias of the disk are most prone to regress” [54].

Patient V., 52 years old, with back pain duration of three days. VAS: 7. The pain extends to the legs, more right, in particular, the anterior-medial parts of the thigh and legs. Hypoesthesia in the dermatome L3, straight leg test 60°. Movements exacerbate the pain.

**Figure 8:**
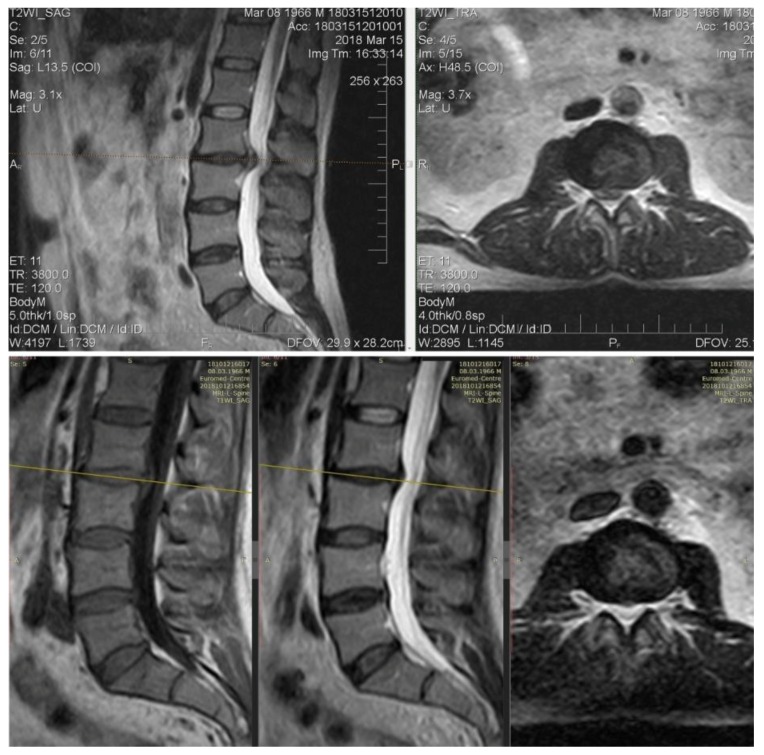
On follow-up T1 and T2-weighted MRI of the lumbar spine, the regressed extruded disc fragment is seen at the L2-3 level and the dural sac is free from compression.

MRI: Extrusion of the intervertebral disk L2–L3 with compression of foraminal channels bilaterally. At day 30, there was a clear reduction in the size of extrusion, which was accompanied by a clinical “recovery”, a complete regression of pain (Figure 8).

No significant difference was found between the effect of dexamethasone or L-lysine treatment with aescinate 10.0 on the course of discopathy (*p* = 0.23). Although in the 10 mL L-lisine aescinate group of L-lysine there were no cases of increase in size or caudal migration of the hernia, the difference with other groups was statistically insignificant, in particular, against the dexamethasone group, where there were three cases of deterioration of morphological characteristics. Additionally, no correlation or modifying effect of the morphometric indices of the intervertebral disc on the pain characteristics of the VAS and BPI, the degree of the straight leg test or the amplitude of the H-reflex (*r* = 0.16), was significant.

## 5. Limitations of the Study

This study is a comparative clinical study of the efficacy of dexamethasone and L-lysine aescinate in pharmacotherapy for back pain. The study did not contain a placebo-control, therefore, it does not allow us to judge the effectiveness of the drugs used compared to the placebo. The research design used allows us to speak only about the similarity or difference in the dynamics of the state of patients receiving different drugs.

Endpoints of the study were the intensity of pain measured on the scale of VAS and the intensity of the straight leg test. Both endpoints may be subject to fluctuations as a result of the patient’s unstable approach to assessing his or her own pain, as well as due to inaccuracies in the measurement of the degree of the straight leg test symptom on the part of the physician and on the patient’s side.

This article also examines the possibility of influencing the assessment of BPI and the degree of the H-reflex to the course of the disease. Both factors are closely related to the intensity of the pain or the symptom of the straight leg test, so it is not possible to correctly isolate their effects from the time-end autocorrelation of the endpoints proven in this article.

## 6. Conclusions

### 6.1. Potential Predictors, Prognostic Factors, and Markers of the Course of Sciatica with Radiculopathy

Our study shows that the size of the intervertebral disk herniation, the degree of the straight leg test, Brief Pain Inventory score, and the H-reflex amplitude does not have a reliable prognostic value and does not allow for the prediction of the course of the radiculopathy, in particular, the regression of pain or need for future surgical treatment. The morphometric characteristics of the intervertebral disc according to MRI do not correlate with the intensity of pain and are not valid predictors of the clinical condition.

### 6.2. Effectiveness of Dexamethasone and L-Lysine Aescinate in Patients with Acute Low Back Pain and Radiculopathy

The lowest levels of pain on the thirtieth day were achieved in patients who received the drug L-lysine aescinate 10 mL, but the decrease in pain was slightly greater in the group administered dexamethasone. Taking into account the side effects of dexamethasone, a solution of L-lysine aescinate can be used to relieve pain symptoms.

The use of a solution of L-lysine aescinate in patients with sciatica and radiculopathy requires further research to more accurately determine the long-term effects and outcome.

## Figures and Tables

**Table 1:** Baseline characteristics. All values are expressed as mean ± standard deviation.

Characteristics	Dexamethasone (*N* = 30)	L-Lysine Aescinate, 5 mL (*N* = 30)	L-Lysine Aescinate, 10 mL (*N* = 30)
Age (years)	48 ± 14.3	46 ± 16.1	49 ± 11.2
No. females	11 (37%)	12 (40%)	10 (33%)
VAS of global pain (0–10)	6.9 ± 1.3	6.1 ± 1.3	5.7 ± 1.7
Straight leg raise angle	42.8° ± 14.2°	52.8° ± 15.6°	50.6° ± 17.7°
Brief Pain Inventory	53.1 ± 9.8	45.3 ± 11.2	46 ± 13.4

**Table 2:** Coefficients of mixed regression model with dependent variable (intensity of pain by VAS) and independent variables: time, the treatment, and estimation of pain by BPI.

Components	Coefficient ± Standard Deviation	*p*-Value
BPI; Time—1st day (correlation with VAS)	β_KOБ−1_ = 0.077 ± 0.026	0.004
BPI; Time—15th day (pain dynamic)	β_KOБ−15_ = −0.021 ± 0.014	0.14
BPI; Time—30th day (pain dynamic)	β_KOБ−30_ = −0.042 ± 0.017	0.018
BPI; L-lysini Aescinate, 5 mL (effect of the drug)	β_KOБ-L-лiз5_ = −0.003 ± 0.031	0.91
BPI; L-lysini Aescinate, 10 mL (effect of the drug)	β_KOБ-L-лiз10_ = −0.028 ± 0.030	0.36
